# Retracted: Correlation of CCNA1 Promoter Methylation with Malignant Tumors: A Meta-Analysis Introduction

**DOI:** 10.1155/2021/2608067

**Published:** 2021-02-02

**Authors:** BioMed Research International

*BioMed Research International* has retracted the article titled “Correlation of CCNA1 Promoter Methylation with Malignant Tumors: A Meta-Analysis Introduction” [1]. This article is one of a series of very similar meta-analyses written by different authors that were published in 2014 and 2015, characterized by the use of particular phrases [2]. The articles have the same structure, with the figures in the same order. The appearance of the figures and parts of the text are also similar.

The article is being retracted with the agreement of the journal and the editorial board due to concerns regarding the reliability of the data. Despite numerous attempts, the authors could not be contacted to provide a response to these concerns.
